# Classification of Arrhythmia in Heartbeat Detection Using Deep Learning

**DOI:** 10.1155/2021/2195922

**Published:** 2021-10-19

**Authors:** Wusat Ullah, Imran Siddique, Rana Muhammad Zulqarnain, Mohammad Mahtab Alam, Irfan Ahmad, Usman Ahmad Raza

**Affiliations:** ^1^Department of Computer Science, Lahore Leads University, Lahore, Pakistan; ^2^Department of Mathematics, University of Management and Technology, Lahore 54770, Pakistan; ^3^Department of Mathematics, University of Management and Technology, Sialkot Campus, Sialkot, Pakistan; ^4^Department of Basic Medical Science, College of Applied Medical Science, King Khalid University, Abha, Saudi Arabia; ^5^Department of Clinical Laboratory Science, College of Applied Medical Sciences, King Khalid University, Abha 61421, Saudi Arabia; ^6^Department of Computer Science, University of Engineering and Technology, Lahore, Pakistan

## Abstract

The electrocardiogram (ECG) is one of the most widely used diagnostic instruments in medicine and healthcare. Deep learning methods have shown promise in healthcare prediction challenges involving ECG data. This paper aims to apply deep learning techniques on the publicly available dataset to classify arrhythmia. We have used two kinds of the dataset in our research paper. One dataset is the MIT-BIH arrhythmia database, with a sampling frequency of 125 Hz with 1,09,446 ECG beats. The classes included in this first dataset are N, S, V, F, and Q. The second database is PTB Diagnostic ECG Database. The second database has two classes. The techniques used in these two datasets are the CNN model, CNN + LSTM, and CNN + LSTM + Attention Model. 80% of the data is used for the training, and the remaining 20% is used for testing. The result achieved by using these three techniques shows the accuracy of 99.12% for the CNN model, 99.3% for CNN + LSTM, and 99.29% for CNN + LSTM + Attention Model.

## 1. Introduction

Cardiovascular diseases (CVDs) are the major public health problem worldwide. Every year almost 17.9 million people waste their lives because of these deadly diseases. Coronary heart disease, cerebrovascular illness, rheumatic heart disease, and other diseases are among the heart and blood vessel disorders known as CVDs. Heart attacks and strokes are responsible for more than four out of every five CVD fatalities, with one-third of these deaths occurring before 70. Willem Einthoven (1860–1927), former professor of mercury electrometer ECG at Leiden University in the Netherlands, has developed mathematical precision. Einthoven published his first paper in 1901 for the galvanometer, which was monitored in 1903 in detail by an ECG new metal survey. In 2002, Willem utilized the ECG for clinical purposes with the help of a string galvanometer [1].

**Figure 1:**
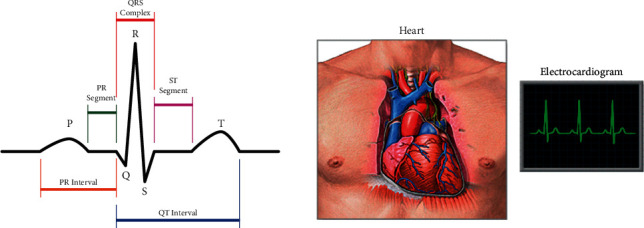
Heart cycle in an ECG.

ECG points P-Q-R-S and T letters to different deflections [2] in Figure 1. Wave and action are summarized in Table 1.

The electrocardiogram (ECG/EKG) is a noninvasive diagnostic technique that records the heart's physiological activity throughout time. Many cardiovascular disorders, such as premature contractions of the atria (PAC) or ventricles (PVC), atrial fibrillation (AF), myocardial infarction (MI), and congestive heart failure, can be diagnosed using ECG data (CHF). The fast development of portable ECG monitors in the medical profession, such as the Holter monitor [3], and wearable gadgets in different healthcare domains, such as the apple watch, has occurred in recent years. Consequently, the analyzed ECG data has risen at a rate that human cardiologists cannot keep up with. So, analyzing the ECG data automatically and correctly becomes an exciting subject. ECG data may also be used for various applications, such as biometric human identification and sleep staging.

**Figure 2:**
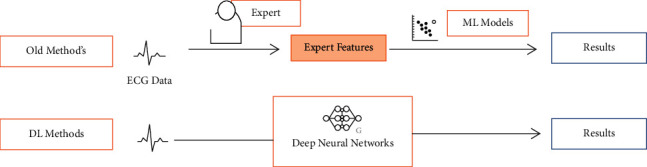
Evaluations between deep learning procedures and old procedures.

Automatic ECG analysis always depends on golden diagnostic principles. The top of Figure 2 presented two-stage methods that define human experts on unprocessed ECG data to use features based on the engineer, which are discussed as “expert features,” and at that point organize choice rules; otherwise different machine learning methods are used to make the final result. As a replacement, feature extraction is made ultimately and automatically using deep learning models based on robust data learning flexible and skills processing structural design. Various studies make sure to experimentally prove that deep learning features are most helpful to expert features used for ECG data [4]. Automatic exposure to ECG-based arrhythmia is very convenient since it eliminates physicians' need to personally interpret the signs and allows people to track their cardiac symptoms using handheld devices. The ECG shows the electrical behavior of the heart over time from electrodes mounted on the scalp, which is the most widely used solution for arrhythmia diagnosis. The ECG leads, which record the heart's electrical potential from various angles and locations, can be used to diagnose disease by searching for irregular waveforms or rhythms. Since cardiovascular diseases have a high death rate, early identification and conclusive distinction of arrhythmias are crucial to medical care [5].

Heartbeats can be usually speedy, or else slow. Heartbeat and extra morphological features (together with spatiotemporal relations between altered genetic factors) can be considered to identify arrhythmia. Some false findings monitored cure can exist. An impression of the altered arrhythmias is happening in this segment. It has to be noted that info is provided, taking into account the average healthy adult. Diagnosis factors differ by gender, race, and age. An example of arrhythmias is given in ECG arrhythmias and their characteristics and results [6, 7]. The traditional approach to diagnosing CVD relies on a patient's medical history as well as clinical trials. Healthcare providers increasingly demanding appropriate medical examinations can be linked to the use of computer-aided diagnosis systems (CADS); [8] DNNs detect arrhythmias in captured ECG signals [9]. The ECG using direct visual inspection is used to look for epileptic form of abnormalities. Most EEG software has a kind of automated imagery detection. During the tremor, however, the epileptogenic neural network has an extremely high electrical activity. This can lead to seizures and fainting complications [10].

Every abnormality from the usual heart rate, including heart rate disorders and regularity or conduction of the cardiac electrical impulse, is called arrhythmias (60–100 beats per minute). The detection of anomalies in heart-based biosignals has attracted considerable interest [7]. The electrocardiogram (ECG) is a nonstationary physiological characteristic that shows the electrical pulse of the heart. When it comes to diagnosing arrhythmia in the recorded ECG signal, contemporary CADS devices use DNNs to decrease the price of continual cardiac supervision [11]. A single-lead ECG signal classification method for arrhythmias is suggested. To achieve this, the system combines three different types of information: RR intervals, signal morphology, and higher-level statistical data. Although the location of the R-wave was artificially distorted by adding jitter, it nevertheless outperformed other approaches in the field [12].

The primary purpose of this study is to contribute to the training stability with the help of a modified deep learning method. A preprocessing approach that significantly enhances the deep learning models' accuracy is suggested for ECG classification. Deep learning methods are used to analyze ECG data. For the analysis of a heartbeat dataset including 1,09,446 beats in five types. The classes included in this first dataset are N, S, V, F, and Q. The second database is PTB Diagnostic ECG Database. The second database has two classes. The techniques used in these two datasets are the CNN model, CNN + LSTM, and CNN + LSTM + Attention Model. This paper is outlined as follows.  Section 2 introduces the related work.  Section 3 describes the material and method of ECG observing systems. It describes contrasts, analyzes each cluster of studies, highlights the significant characteristics, and discusses specific research challenges.  Section 4 describes the result and summarizes it.  Section 5 contains the discussion.  Section 6 gives the conclusion of the article.

## 2. Related Works

Murat et al. [5] presented a review for knowledge, and more study on deep learning models became a common way for ECG data to be classified. They function ECG data from 5 groups of 100,022 beats as of the MIT-BIH rhythmic database to test the literature's most used profound learning strategies. Hong et al. [4], the writer of a survey paper, explains a systematic analysis of ECG data deep learning methods on or after the modeling perspectives. The writer applied ECG deep learning models published by Google Scholar, PubMed, and Digital Bibliography & Library Project between 1 Jan 2010 and 29 Feb 2020. Ebrahimi et al. [9] explored many DL methods, including CNN, DBN, recurrent neural network, short-term memory (LSTM), and gated recurrent machine. The majority of papers reviewed used at least one form of DL technique to extract and classify the features. Acharya et al. [10] found general and predictive classes with 13 deep layers of a fully convolutional neural network (CNN). This approach can be time consuming, limited by a practical artifact, and responsible for flexible results at the professional level of students. Like the ANN, the final CNN model performance judgment is dependent on the network structure weights and preferences of the previous layers. The pooling process decreases the size of the output neurons of the coevolutionary layer to decrease the calculation amplitude and avoid overfitting. The suggested method's accuracy, specificity, and sensitivity were 88.67%, 90.00%, and 95.00%, respectively.

Acharya et al. [10] focus on the diverse ways to diagnose myocardial infarction (heart attack) and distinguish arrhythmias (heartbeat variations), hypertrophy (increased heart muscle thickness), and heart enlargement. It is concluded that fuzzy-based technology is successful in the analysis of computerized ECG but needs more research. The precision of the definition is 86.67%, while the specificity is 93.75%. The built model is well tested with various performance metrics but can be further modified for practical applications. Limaye and Deshmukh [13] discussed that the ECG signal concept consists of very low-frequency signals to about 0.5–100 Hz. Cardiac monitors are the instruments that enable the ECG recording to be filtered. The Low Pass Filter (LPF) is applied to eliminate the unwanted high-frequency noise signal. Mbachu et al. [14] introduced LPF, HPF, and BSF architecture with the window Kaiser, where these three filters are related serially, processing the signal within the range of 0 to 100 Hz and attenuating a signal of 13 dB for each filter in order of 200 and with interference signals. Dias et al. [12] introduced a digital notch filter design using the Hamming Window, with a 50 Hz interference effect, resulting in 13.4 dB attenuation.

Litjens et al. [15] studied more than 80 articles covering modals of intravascular visual cohesiveness tomography and echocardiography from cardiac magnetic resonance, computed tomography, and single-photon computed tomograms. It explains the basic principles that underlie the most efficient deeper learning algorithms. Kaplan Berkaya et al. [16] presented a survey paper on ECG signal and studied 1538 records, including heart rate measurements, cardiovascular function, cardiac disorders diagnosis, emotion detection, and biometrical identification. They also summarized the most advanced research on the preprocessing phase in ECG signals analysis. The study of their results under different preprocessing procedures is of great significance. Haroon [17] represented the meaning of deep learning approaches, such as convolution neural network (CNN) based algorithms, which may prevent manual ECG signal functionality. The Python PTB and MIT-BIN Data Set ECG database (wfdb) library were used for study, and various features and data variations were made. During the classification of these time series signals, different methods were developed for applying machine learning algorithms. For deep-seated convolution, transfer training, and computation in the Google Colab setting, multilayer CNN, ResNet-50, and VGG-16 were used. The result is CNN = 83.1%, ResNet-50 = 83.5%, and overall accuracy = 99%.

Type [18] studied common characteristics of medical and engineering cases related to ECG diagnosis. It includes the recent breakthrough deep learning data classification, deep neural network systems for wearable ECG monitors, and automated cloud health platform detection. The main components of system hardware are a monitor system and an android terminal. Serhani et al. [19] discussed a basic model for ECG monitoring systems that should be set up and problems identified. They also highlighted the value of intelligent surveillance systems that exploit emerging technology to provide *e* client, cost-aware, and fully integrated monitoring systems, including in-depth learning, artificial intelligence (AI), big data, and the IoT. Naik et al. [20] defined the governance ECG impulses should be registered and tracked to detect rhythmic cardiac issues. A nonlinear compression structure with a coevolutionary automatic encoder (CAE) is applied to reduce the rhythmic beats signal scale. The accuracy is overall 99.0%. Jiang et al. [21] solved the difficult disequilibrium in the ECG classification of electric cardiograms by a new MMNNS. The contrasts with other state-of-the-art approaches on three datasets use standard parameters to reveal the dominance of the new system. The overall accuracy was 96.6 percent, and the MAUC score was 0.978. Han and Shi [22] introduced a novel method of detecting and locating the MI that incorporates a multilead residual neural network structure (ML-ResNet) with 3 residual blocks and attributes fusion across 12-lead ECG records. Experimental results based on the PTB database indicate that our model produces superior 95.49% precision results.

Hao et al. [23] proposed a multibranch MI fusion framework for automated MI screening of 12-lead ECG images. The Zhejiang Second People's Hospital of China dataset presented 95712-lead ECGs, consisting of 483 MI images and 474 non-MI images. The result accuracy is 94.73%, sensitivity 96.41%, specificity 95.94%, and F1-score 93.79%. Kuznetsov and Moskalenko [24] developed a vibrating car encoder to generate an ECG signal for a heart cycle. The new features derived lead to the quality enhancement of cardiovascular diagnosis automatically. Shaker et al. [25] discussed two types of problems utilizing GAN, which have two neural networks such as generator and discriminator, one competing against the other. By increasing the initial imbalanced dataset using the proposed techniques, the efficiency of the ECG classification can be increased more efficiently than using the same techniques just trained in the original dataset. The overall accuracy is 98.0%, precision 90.0%, specificity 97.4%, and sensitivity 97.7%. In the study of Huang et al. [26], the time domains of ECG were defined by a short-time Fourier transformation, which included five forms of heartbeat normal beat (NOR), left bundle branch block beat (LBB), premature ventricular contraction beat (RBB), and atrial premature contraction beat. The average accuracy is 90.93%.

Huang et al. [27] presented a precise classification system based on the intelligent ECG classification with the aid of fast residual convolutionary neural networks (FCResNet) proposed to promote smart classification of arrhythmia with high precision, averaged 98.79% precision when set to 2. 20 batch size parameters and low-frequency subspaces were chosen in MOWPT as the classifier. The system was tested on a patient's ECG. Naik [20] utilized a multirate cosine filter banking architecture to assess the ECG signal coefficients in different subbands. The findings suggest that 99.40%, 98.77%, and 100% were successful in detecting an FN-dependent total volume of cosine based on factors of AF. Avanzato and Beritelli [28] had given a solution to the advancement of automatically diagnosed heart attack systems. The proposed neural architecture is built on the recent success of the CNN network (CNN) and other networks. It could be used in a new way of detecting heart attacks, with 98.33% mean accuracy, 98.33% sensitivity, 98.35% specificity, 1.65 percent false-positive ratio, 1.66% false-negative ratio, and 98.33% F1-score.

## 3. Material and Method

To assess deep learning techniques frequently utilized in the literature, we analyzed ECG data from five separate classes containing 109,446 beats collected from the MIT-BIH arrhythmia database and the PTB Diagnostic ECG Database. The results were evaluated using various applications, ranging from the most basic to the most advanced.

### 3.1. ECG Dataset

This dataset is divided into two sets of heartbeat signals obtained from the MIT-BIH Arrhythmia Dataset and the PTB Diagnostic ECG Database, two well-known datasets for heartbeat classification. To assess deep learning models, we used a dataset with a sampling frequency of 125 Hz with a total of 109446 ECG beats. The major classes of N, S, V, F, and Q are included in the MIT-BIH arrhythmia database, and the PTB Diagnostic ECG Database has two classes. Both sets include a sufficient amount of data to train a deep neural network. This dataset has been used to investigate heartbeat categorization using deep neural network architectures and test specific transfer learning capabilities. For the typical cases afflicted by various arrhythmias and myocardial infarction, the signals correspond to electrocardiogram (ECG) forms of heartbeats.

These signals are segmented and preprocessed, with each segment representing a heartbeat.

- Regular, right, or left bundle branch block, nodal escape, and atrial escape are all in the “N” category
- Atrial premature, aberrant atrial premature, nodal premature, and supraventricular premature fall under the “S” category
- Ventricular escape and premature ventricular contraction are seen in the “V” category
- Fusion of ventricular and normal is labeled as an “F” class
- Paced and fusion of paced and normal unclassifiable are labeled as a “Q” class

The basic deep learning models for heartbeat detection and more sophisticated deep learning models for cardiac identification are based on networks. The investigations were conducted using deep learning techniques.

They are specified for heartbeat analysis, and some of these approaches were applied to cardiac datasets, and the findings were then assessed.

### 3.2. Implementation

In the implementation of this work, we utilized NumPy Community [29] and McKinney and Team [30] with Imambi et al. [31] and Seaborn [32] for python backend deep learning library to implement deep learning techniques. Google colaboratory [33] was used to train the model. Raw ECG signals were scaled in the 0–1 range before being standardized. The test data findings were evaluated using the accuracy, recall, precision, and F-Score performance measures.

### 3.3. Methodology

A thorough study has been carried out in this section for classes and the number of beats in Table 2.

We used a total of 10 .csv files and 3. pth files in which total data contains 10505 rows and 188 columns

**Figure 3:**
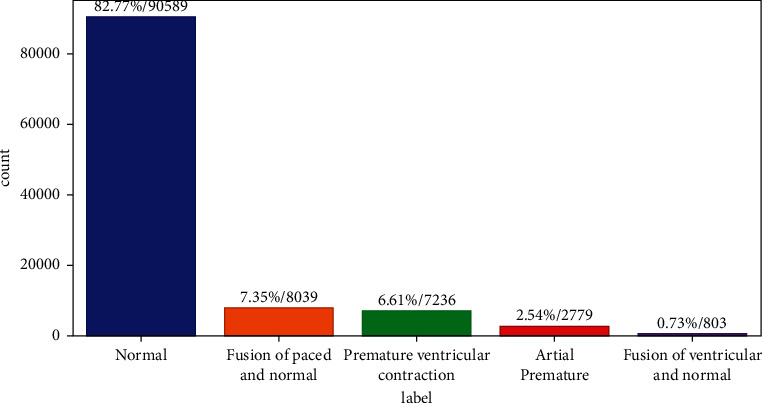
Label, number of data, and data percentage.

Figure 3 shows the normal percentage is 82.77, the fusion of paced and average percentage is 7.35, premature ventricular contraction percentage is 6.61, atrial premature percentage is 2.54, and fusion of ventricular and average percentage is 0.75 data, using Generative Adversarial Network

We can see that the generator creates primarily dominant signal types because this is a standard procedure for training a GAN model. We had a total of 803 signals in the “fusion of ventricular and normal” class, the majority of which are pretty similar, and this is what the GAN model learned to create.

**Figure 4:**
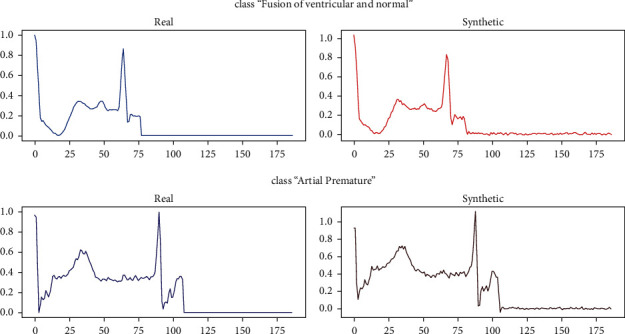
Real and synthetic signals.

**Figure 5:**
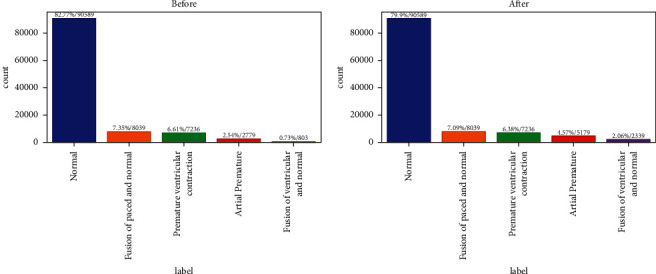
Label, number of data, and data percentage after GAN.

After using GAN, Figure 4 shows the adjustment and change that occurred in the forms of beats.  Figure 5 shows the difference between the results achieved before and after using GAN aptly.

Figure 5 shows the normal percentage is 79.9, the fusion of paced and average percentage is 7.09, premature ventricular contraction percentage is 6.38, atrial premature percentage is 4.57, and fusion of ventricular and average percentage is 2.06 data, the result of GAN (Generative Adversarial Network).

We used a GAN repository with code to generate new artificial data for classes with little data, and now the dataset looks like this.

**Figure 6:**
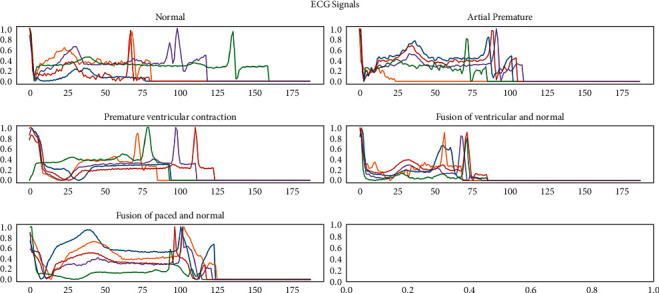
ECG signal.

The waveforms of ECG signals from all five collections contrast the dataset used in [34] as in Figure 6. These alterations are performed to each signal in the dataset. Each of these alterations is stored and added to the actual dataset, resulting in a total of 2,79,149 samples out of 16,372,411 unique values in the whole dataset. These signal modifications are lossless [35] and do not affect the signal's nature, standard, or file size. Each layer in deep learning studies a specific function that our deep learning model can extract.

### 3.4. Proposed Model

**Figure 7:**
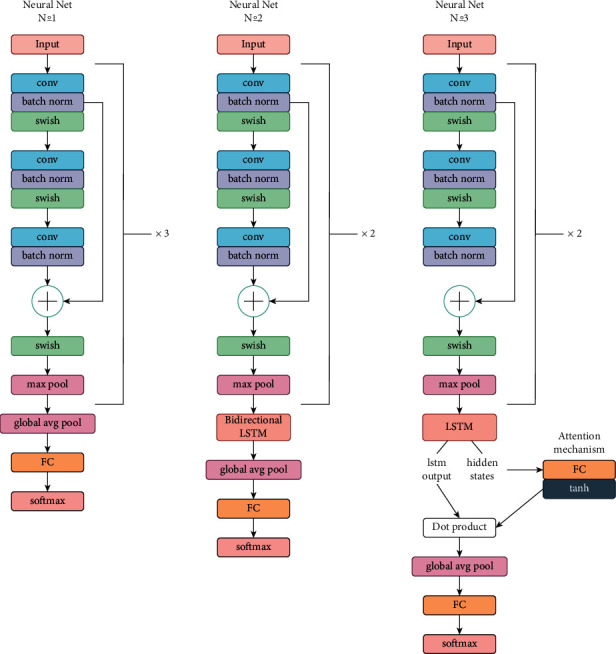
Proposed architecture.

In ECG classification, we will employ the attention method to “clarify” key characteristics in Figure 7, such as recurrent or convolutional layers. The attention mechanism is best taught using the seq2seq model as an example; therefore reading this interactive would be a fantastic idea.

**Figure 8:**
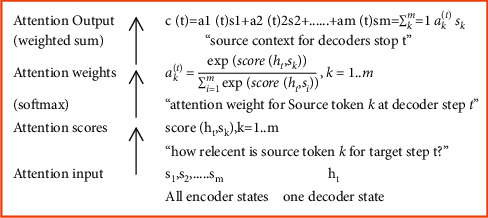
General computing scheme.

Following are the decoder steps used in Figure 8:

- Get attention information from all encoder states *s*1, *s*2, ... *s*k, as well as a decoder state ht.
- Calculate attention levels.
- Sk attention calculates the “relevance” of each encoder state for this decoder state ht. It uses an attention function that takes one decoder state and one encoder state and returns the scalar value score (*h*_*t*_, *s*_*k*_).
- A probability distribution softmax applied to attention ratings computes attention weights.
- Compute the weighted sum of encoder states with attention weights as an attention output.

**Figure 9:**
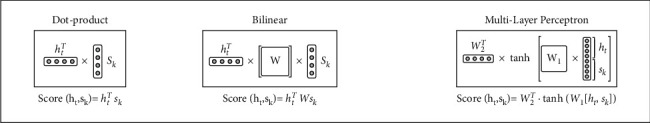
Calculation of attention scores.

The following are the most common methods for calculating attention scores in Figure 9:

- The easiest way is to use a dot-product
- Practical approaches to attention-based neural machine translation employed the bilinear function (“Luong attention”)
- The approach presented in the original study was the multilayer perceptron (also known as “Bahdanau attention”)

**Figure 10:**
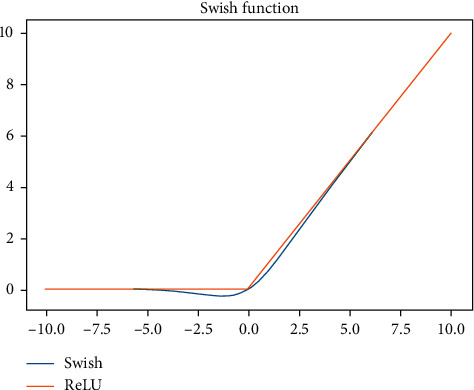
Flowchart of Swish function.

We used two functions, ReLU and Swish, in Figure 10. Rectified linear unit (ReLU) is a transfer or activation function. It helps the neural network decide whether or not to output yes or no by mapping output to values such as 10 and 0 or −10.0 and 10.0, depending on the model function. Swish is a nonmonotonic, smooth function that regularly equals or exceeds ReLU on deep networks in several complex areas such as image classification and machine translation. It is unbounded above and below, and it is the nonmonotonic characteristic that makes the distinction. In a self-gating situation, just a single scalar input is required, but numerous two-scalar inputs are required in a multigating scenario. It was motivated by the LSTM's usage of the sigmoid function.

## 4. Results

Experiments were carried out using our improved dataset and the original dataset used in the proposed model. The three sets of findings generated are an initial model with the original dataset, an initial model with an augmented dataset, a newly recommended model with the original dataset, and a new proposed model with an improved dataset.

**Figure 11:**
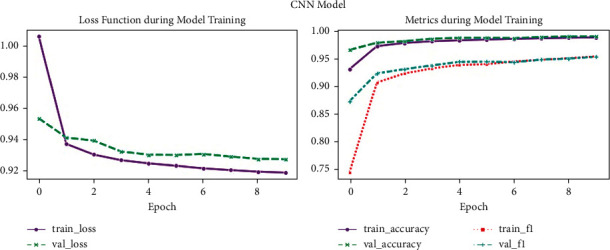
CNN model performance.

Figure 11 shows loss function during model training and metrics during model training (CNN model). Graphs depict CNN network performance on the heartbeat dataset during the training phase: (a) loss values (training and validation) and (b) graphs of accuracy (training and validation).

**Figure 12:**
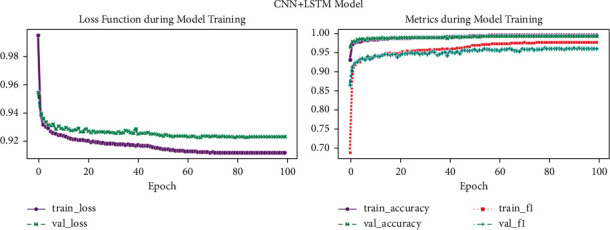
CNN and LSTM model.

Figure 12 shows loss function during model training and metrics during model training (CNN + LSTM model). Graphs depict CNN and LSTM network performance on the heartbeat dataset during the training phase: (a) loss values (training and validation) and (b) graphs of accuracy (training and validation).

**Figure 13:**
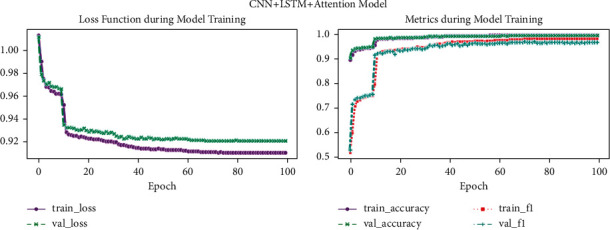
CNN + LSTM + Attention Model.

Figure 13 shows loss function during model training and metrics during model training (CNN + LSTM + Attention Model). Graphs depict CNN + LSTM + Attention Model performance on the heartbeat dataset during the training phase: (a) loss values (training and validation) and (b) graphs of accuracy (training and validation).

**Figure 14:**
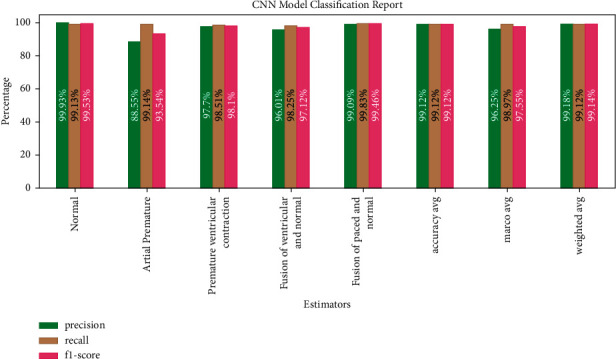
CNN model classification report.

Figure 14 shows CNN model classification with 99.12% average accuracy (precision, recall, and *F*1-score value are equal).

**Figure 15:**
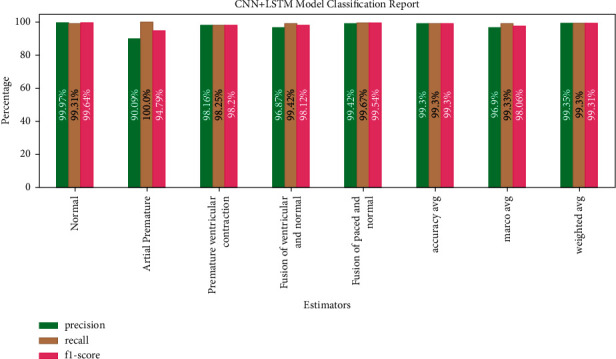
CNN + LSTM model classification report.

Figure 15 shows CNN + LSTM model classification with 99.3% average accuracy (precision, recall, and F1-score value are equal).

**Figure 16:**
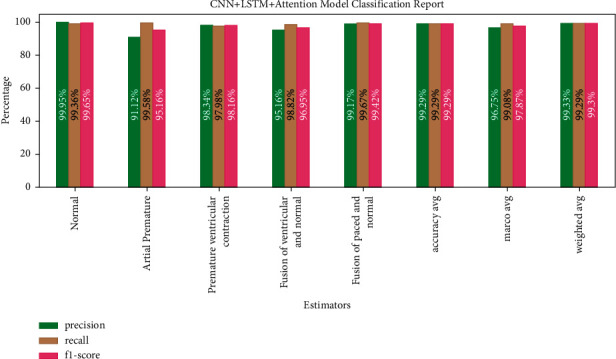
CNN + LSTM + Attention Model classification report.

Figure 16 shows CNN + LSTM + Attention Model classification with 99.29% average accuracy (precision, recall, and *F*1-score value are equal).

**Figure 17:**
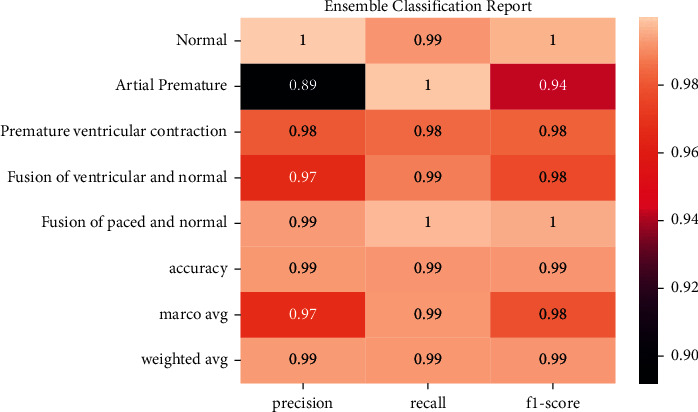
Ensemble classification report.

Figure 17 shows the report ensemble classification report accuracy (precision, recall, and *F*1-score).

## 5. Discussion

This part discusses the classification of cardiac/heartbeat that seems to be best known in the forms of conditions. Another common area of application is the heartbeat style identification to separate different ECG beats from each other. The more recent areas of use of ECG research are biometric detection and emotional recognition. The initiative for the early detection of diseases is a famous study and classification. The issues of biometric authentication and the application of emotional recognition can be resolved by various techniques, unlike heartbeat type detection.

We looked at literature publications that used deep learning on arrhythmia ECG data in this investigation. The following are some key observations made as a result of these investigations.

The imbalance of the ECG dataset is a significant issue because certain classes have a lot of data relative to others, which might lead to false information about model performance. Some researchers have devoted their attention to this issue and suggested remedies.

CNN modeling has been the subject of a lot of recent studies in this field. Both representation and sequence characteristics of ECG data increase classification performance in our experiments. As a result, effective hybrid models can extract more distinguishing characteristics from ECG data.

Another intriguing use in this subject is using separate models for shallow categorization while using deep models as feature extractors. The benefits of shallow categorization may be taken advantage of with this method.

When performed on databases with vast volumes of high-quality data, deep learning models perform well. As a result, a study on newly created big ECG datasets might lead to more effective models.

Using the deep learning method, some recent ECG classification experiments are presented. When this study is compared, it becomes clear that CNN models outperform alternative approaches. Aside from the challenges in designing and adjusting CNN models, the high computational cost of neural networks is the most significant drawback. Another disadvantage is that they require a large dataset for successful training. The main benefit of ECG databases is that there are many databases and a large number of signal situations. Accuracy, precision, recall (or sensitivity), F-measure, and correlation coefficient success indicators for ECG analysis and classification can be mentioned which are defined as follows: accuracy = TP+TN/TP+TN+FP+FN, precision = TP/TP+FP, recall = TP/TP+FN, and F-measure *F*_1_ = (recall^−1^+precision^−1^/2)^−1^=2*∗*(precission*∗*recall/precision+recall).

## 6. Conclusion

In this article, we examined and assessed the deep learning techniques used to classify a heartbeat. We improved the model accuracy by scrutinizing the datasets. Because the proposed model includes ten residual blocks, there is a possibility of overfitting the data. Naturally, the enlarged dataset makes it harder to classify data during the testing phase because of the human factor. However, the suggested model still has a high level of accuracy. This demonstrates its ability to produce very accurate predictions with a 99.12 percent accuracy rate for the CNN model, 99.3 percent accuracy for the CNN + LSTM model, and 99.29 percent accuracy for CNN + LSTM + Attention Model. In the future, this study should be conducted in binding domains like cloud and mobile systems. It is also vital to develop wearable technologies with integrated low-power consumption wearable technologies.

## Figures and Tables

**Table 1:** Wave and action [2].

Wave	Action
P	Depolarization of the atria
Q	Activation of the anteroseptal region of the ventricular myocardium
R	Depolarization of the ventricular myocardium
S	Activation of the posterior basal portion of the ventricles
T	Rapid ventricular repolarization

**Table 2:** Beat types and classes.

Classes	Beat type	Number of beats
*N*	Normal	90589
*S*	Fusion of paced and normal	8039
*V*	Premature ventricular contraction	7236
*F*	Atrial premature	2779
*Q*	Fusion of ventricular and normal	803

## Data Availability

The data that support the findings of this study are available from the corresponding author upon reasonable request.
